# 3D and 4D printing hydroxyapatite-based scaffolds for bone tissue engineering and regeneration

**DOI:** 10.1016/j.heliyon.2023.e19363

**Published:** 2023-08-22

**Authors:** Sina Soleymani, Seyed Morteza Naghib

**Affiliations:** Nanotechnology Department, School of Advanced Technologies, Iran University of Science and Technology (IUST), Tehran, Iran

**Keywords:** 3D printing, Hydroxyapatite, 4D printing, Polymer, Scaffold, Bone tissue engineering

## Abstract

The osseous tissue can be classified as a nanocomposite that encompasses a complex interweaving of organic and inorganic matrices. This intricate amalgamation consists of a collagen component and a mineral phase that are intricately arranged to form elaborate and perforated configurations. Hydroxyapatite, whether synthesized artificially or obtained from natural sources, has garnered considerable attention as a composite material in the field of bone tissue engineering due to its striking resemblance to bone in terms of structure and characteristics. Hydroxyapatite (HA) constitutes the predominant ceramic biomaterial for biomedical applications due to its ability to replicate the mineral composition of vertebrate bone. Nonetheless, it is noteworthy that the present biomimetic substance exhibits unfavorable mechanical characteristics, characterized by insufficient tensile and compressive strength, thus rendering it unsuitable for effective employment in the field of bone tissue engineering. Due to its beneficial attributes, hydroxyapatite (HA) is frequently employed in conjunction with various polymers and crosslinkers as composites to enhance mechanical properties and overall efficacy of implantable biomaterials engineered. The restoration of skeletal defects through the use of customized replacements is an effective way to replace damaged or lost bone structures. This method not only restores the bones' original functions but also reinstates their initial aesthetic appearance. The utilization of hydroxyapatite-polymer composites within 3D-printed grafts necessitates meticulous optimization of both mechanical and biological properties, in order to ensure their suitability for employment in medical devices. The utilization of 3D-printing technology represents an innovative approach in the manufacturing of HA-based scaffolds, which offers advantageous prospects for personalized bone regeneration. The expeditious prototyping method, with emphasis on the application of 3D printing, presents a viable approach in the development of bespoke prosthetic implants, grounded on healthcare data sets. 4D printing approach is an evolved form of 3D printing that utilizes programmable materials capable of altering the intended shape of printed structures, contingent upon single or dual stimulating factors. These factors include aspects such as pH level, temperature, humidity, crosslinking degree, and leaching factors.

## 1 Introduction

The investigation into the application of HA in the field of bone tissue engineering remains an active area of exploration. The preliminary results entailed the production of synthetic HA. Subsequently, HA sourced from natural materials was unearthed and advanced in biomedical contexts, such as bone restoration [1]. Belonging to the family of calcium phosphates, Hydroxyapatite (HA) is acknowledged as a bioceramic, much like its counterparts. Among its distinct properties, HA exhibits superior thermodynamic stability and is second only to Fluorapatite in terms of solubility under physiological conditions. Furthermore, it is quite noteworthy that HA mirrors the structural and functional aspects of biominerals such as teeth and bone closely. This correlation, thus, makes HA a crucial research subject for addressing bone and dental anomalies [2]. Hydroxyapatite (HA) is known for its bioactivity, biocompatibility, osteoconductive capabilities, and lack of immune response-these are proven and expected characteristics due to its chemical similarity to biological forms. Its easy manufacturing methods, reasonable price, and fundamental features make HA a suitable material for the creation of implants and scaffolds. It can also be used as a targeted drug carrier for a variety of bone diseases [3]. Subsequently, inadequacies in the structural integrity of HA were identified, prompting a restorative process that entailed discernments pertaining to the manipulation of HA fabrication techniques, as well as the integration of alternate elements by means of substitution or doping. The integration of several constituents, including biomaterials, within bone tissue engineering materials, presents an auspicious direction in the realm of bone reparative therapeutics. HA, as a tissue engineering material, bears resemblance to the minerals found in bone and teeth. The aforementioned tested material exhibited favorable characteristics of bioactivity, biocompatibility, as well as osteoconductive. The functional potential of the specified entity encompasses its capacity to fulfill roles as a bone filler, an implant, and a bone scaffold. The optimal performance, mechanical properties, and biocompatibility of HA is reliant upon a collaborative relationship with additional metals, minerals, and collagen supports [1,4,5].

Enhancing the biological and biochemical properties of HA is essential for its effective utilization in bone tissue engineering [6]. Despite the utilization of natural constituents like fish, eggs, shellfish, and other analogous materials, there remains a requisite for enhancement in both mechanical and biological properties [7].

The utilization of HA as a coating in bone implants is a prevalent practice aimed at bolstering the strength of the implant material. Nevertheless, HA finds application in the field of implantation, where it acts as a composite material with additional reinforcement substances [[8], [9], [10]]. The utilization of synthetic materials in implants confers significant advantages for bone repair; however, the utilization of biomaterials, particularly HA, entails superior biomimetic characteristics in relation to structural, functional, and performance aspects when interacting with human bones [11].

The utilization of HA as an implant material has revealed several deficiencies, predominantly in its susceptibility to damage. Consequently, there exists a pressing need to augment its resilience by exploring alternative material combinations and identifying optimal fabrication methodologies [12]. Recent studies have not limited themselves to HA coatings alone, opting for other combinations as well to enhance the mechanical, antibacterial, and osseointegration features of implants [[12], [13], [14]].

In examining the benefits of HA, its third-generation form plays a vital role as an advanced drug carrier. This complex, synthesized version of HA can specifically target injured bone areas, allowing for more precise delivery of intended therapeutic agents, such as stem cells. The bio ceramic properties inherent in HA facilitate physical and chemical interactions with drug molecules. This promises controlled release in a preferred time scale, optimizing treatment effectiveness [[15], [16], [17]]. For instance, HA-Chitosan demonstrates a unique polycationic quality due to the incorporation of amino groups in its CS backbone. This characteristic facilitates the encapsulation or absorption of negatively charged organic compounds. Moreover, this polysaccharide exhibits inherent bio adhesion, biodegradability, and modifiability, making it an excellent candidate for the creation of an effective drug delivery vehicle. This vehicle can control drug release and boost bioavailability, thanks to the inherent properties of its biomaterial component [18].

Additive Manufacture (AM) serves as the underlying technique for both 3D and 4D printing technologies [19]. The microstructures produced through 3D printing exist in a stationary state. This limitation can be addressed by utilizing four-dimensional (4D) printing, where a sophisticated spontaneous structure is created that can change with time, reacting in a specific way to outside stimuli. 4D printing is an evolution of 3D printing, providing enhanced capabilities beyond its predecessor. Despite 4D printing being chiefly rooted in 3D printing and becoming a subset of additive manufacturing, the structures produced move beyond being unchanging. Through the application of external stimuli, these objects can metamorphose into intricate structures, modifying their size, shape, characteristics, and functionality [20]. This dynamic aspect brings a sense of life and activity to the otherwise stationary world of 3D printing.

## 2 Application of 3D printer in tissue engineering

The field of tissue engineering necessitates a comprehensive comprehension of the intricate biological mechanisms involved in cellular proliferation and differentiation [[21], [22], [23], [24]]. The initial stage of tissue engineering typically involves the implementation of a scaffold, a crucial three-dimensional framework that enables the proper proliferation and differentiation of cells that are either embedded within, or infiltrating, said scaffold [[25], [26], [27], [28]]. A range of conventional techniques are employed in scaffold production, including solvent-casting particulate-leaching, gas foaming, fiber meshes/fiber bonding, phase separation, melt molding, emulsion freeze drying, solution casting, and freeze drying. These techniques are extensively elucidated in other academic literature [29,30]. The traditional techniques in use possess several limitations, as they are frequently insufficient in generating accurate pore size, pore geometry, as well as attaining high degrees of interconnectivity and mechanical strength [29,30]. The burgeoning technique of three-dimensional (3D) printing technology has shown significant potential in the production of scaffolds with exceptional levels of precision and accuracy [[31], [32], [33]]. This capability permits the fabrication of complex, biomimetic 3D structures featuring intricate details [30].

The strategies as of now being utilized to realize 3D printing of platforms include a step-by-step process, which incorporates, but isn't constrained to, coordinate 3D printing, intertwined statement modeling, and specific laser sintering [34,35]. These strategies have been employed to provide platforms that encompass systems ranging from millimeter to nanometer-scale structures [36]. Another hindrance involves the elapsed time required for platform fabrication, which progressively increases in tandem with the precision and complexity of the framework design [37].

Tissue engineering with 3 d printer experts empowered to create scaffolds with the ability to emulate the intricate arrangements of the extracellular matrix (ECM), thus instilling a microenvironment conducive to cell adhesion, propagation, dispersion, and specialization, with the prospective capacity to manifest practical bodily tissues [36].

When constructing scaffolds that are appropriate for their intended purpose, it is paramount to take into account essential factors such as biocompatibility, biodegradability, pore interconnectivity, pore size, porosity, and mechanical properties. The properties of biocompatibility and biodegradability are of prime importance for scaffold materials as they ensure the degradation of these materials into non-toxic substances while preserving the desired living tissue. Moreover, it is essential for the material to elicit negligible inflammatory reactions, consequently diminishing the probability of rejection by the recipient's safe framework. It would be advantageous if scaffold materials could function as substrates that facilitate cellular attachment, proliferation, and differentiation. In light of cellular proliferation and differentiation, it is crucial that a scaffold possess the capacity to endure forces exerted by the cells; otherwise, its structural failure would culminate in inadequate diffusion of oxygen, nutrients, and waste and, as a consequence, inefficient formation of tissue. Ultimately, the mechanical stability of a scaffold must be structurally robust in order to endure regular bodily movement and activities [38].

In order to attain biomimicry of the extracellular matrix (ECM), scaffolds must possess a host of characteristics that include biological activity, superior mechanical properties, facile processability, and controlled rates of degradation. In the generation of intricate scaffolds, it has become increasingly common to employ hybrid systems that incorporate a blend of synthetic and natural polymers [[39], [40], [41]].

It's important to note that 3D-printed HA scaffolds have proven to be effective delivery mechanisms for osteogenic growth factors, such as bone morphogenetic protein-2. This capability enhances in vivo bone regeneration [42]. Nanomaterials and nano-structure is very excellent and novel Nanomaterials and nanostructures advanced materials in the medicine [[43], [44], [45], [46]] and industries [[47], [48], [49]]. Reports indicate that the application of nanosized hydroxyapatite (HA) particles in 3D-printed bone composite scaffolds, specifically poly-caprolactone (PCL) blended with nanosized HA particles, boosts adhesion, viability, and osteogenic differentiation of human mesenchymal stem cells (hMSCs). This performance is superior compared to similar scaffolds incorporating microsized HA particles [50].

## 3 Methods used to make hydroxyapatite scaffolds with 3D printer

### 3.1 Fused deposition modeling (FDM)

The Fused Deposition Modeling (FDM) technique is widely adopted in 3D printing, where a thermoplastic polymer is melted and extruded from a nozzle to construct a three-dimensional object by additive layering. In the context of hydroxyapatite-polymer composites, the incorporation of hydroxyapatite (HA) particles into the polymer matrix is achieved through the process of mixing, followed by feeding the resulting mixture into the extruder of a printer. The Fused Deposition Modeling (FDM) technique is a cost-effective manufacturing technique typically characterized by its simplicity. However, its application can be limited when higher fidelity structures need to be produced due to the constraints of the extrusion process [51,52].

Achieving an FDM-compliant composite material of superior grade necessitates that the filaments possess superior stiffness, as well as low melting viscosity, which both ultimately rely on appropriate powder dispersion [53].

### 3.2 Stereolithography (SLA)

The Selective Laser Ablation (SLA) technique is a three-dimensional (3D) printing methodology that leverages the use of a laser to selectively solidify a liquid resin. In the context of HA-polymer composites, the incorporation of HA particles into the resin matrix is achieved via a laser-assisted printing process, whereby the particles are cured layer by layer during the fabrication of the final structure. The employment of Stereolithography (SLA) has shown potential in generating intricate structures at a resolution of high fidelity. However, the rigidification of the resin may lead to brittleness and the occurrence of fractures [[54], [55], [56]].

**Fig. 1:**
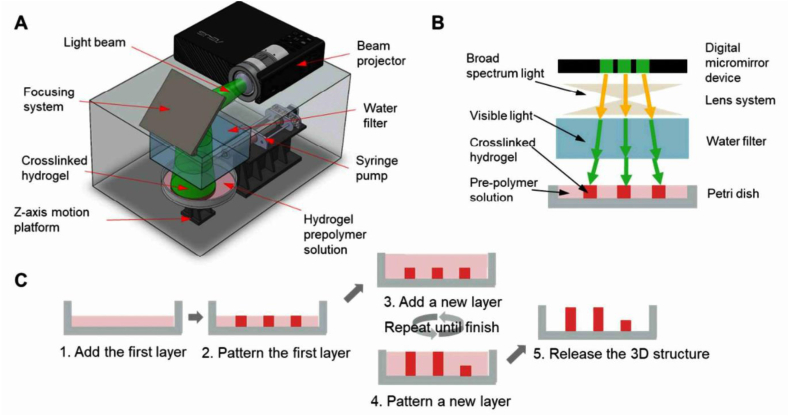
A schematic diagram of the visible-light-based Stereolithography Apparatus (SLA) 3D printing system. Figure (A) illustrates the various components involved in the SLA printing system. Additionally, figure (B) provides insight into the working principles underlying single-layer printing, while the multiple layer printing process is depicted in figure (C) multiple layer printing process [59].

Fig. 1 depicts a schematic diagram of the visible light-based stereolithography (SLA) 3D printing system. Fig. 1a shows the different components involved in the SLA printing system. This figure visually shows the setup and layout of the different parts of the SLA system. In addition, Fig. 1b provides insight into the underlying working principles of single-layer printing, while the multilayer printing process is illustrated in Fig. 1c. The utilization of printing systems employing visible light was exhibited by Woesz and colleagues [57]. Microporous HA scaffolds were produced through the employment of the stereolithography apparatus (SLA) technique that utilizes visible light. The resulting scaffold exhibits a strut size of 450 μm and encompasses interconnectivity via premeditated macrostructural porosity [57]. A further investigation was conducted by Chen and colleagues [58]. The methodology of selective laser sintering (SLS) was utilized in the manufacture of a HA composite scaffold, with the evaluation of the biocompatibility of the resultant resin. The present study elucidated that the utilization of photosensitive resin in the production of SLA prepared HA scaffold led to the manifestation of toxic effects. The results of the study indicate that the photosensitive resin undergoes complete pyrolysis during the scaffold preparation process. The resulting HA material exhibits micro holes and is assessed to possess good biosafety properties during pre-experimental evaluation involving rabbit parietal implantation [58].

### 3.3 Selective laser sintering (SLS)

SLS is a 3D printing technique which involves the targeted utilization of a laser to indiscriminately amalgamate powdery substances. HA-polymer composites are produced by blending HA particles with polymer powder and introducing the resulting mixture into the build chamber of the printer. The laser operates in a precise manner by melting only the polymer powder and integrating it with the HA particles in order to gradually form a layered 3D structure. The utilization of selective laser sintering technology has resulted in the production of robust and long-lasting structures; however, this manufacturing process incurs significant costs and requires a considerable amount of time to accomplish [60,61].

### 3.4 Inkjet 3D printing

The Inkjet 3D printing technique involves the utilization of a specialized printing head to dispense tiny droplets of material onto a designated build platform. In the HA-polymer composite context, the HA particles are immersed in a liquid polymer solution and subsequently introduced into the printer head. The printer's head proceeds to deposit droplets of the solution onto the build platform, subsequently undergoing a curing process utilizing UV radiation. The utilization of inkjet 3D printing presents a comparatively expeditious and cost-effective technique, though may not prove to be optimal when aiming to print voluminous or intricate structures [[62], [63], [64]].

The process of 3D printing, utilizing inkjet technology, comprises two distinct modes of operation, which are generally referred to as continuous inkjet printing, producing a continuous stream of liquid droplets, and drop-on-demand inkjet printing, generating singular droplets [65].

## 4 4D printing and applications in tissue engineering

The proposal for the concept of 4D printing was initially made by professor Tibbits in 2013 [66]. Smart materials are utilized to construct 3D microstructures which have the ability to change in a preset way as time progresses. This concept has led to the emergence of a novel term - namely, “4D printing” [67]. Prof. Tibbits has characterized 4D printing as an advanced design of a complex structure. This structure metamorphoses with time due to environmental interaction, indicating the birth of the 4D printing concept. He originally formulated the definition of 4D printing with the equation, “4D printing = 3D printing + time”. This equation implies the evolving shape, structure, or functionality of 3D printing over time [66,68,69]. According to Zhong and colleagues, 4D printing is articulated as the Additive Manufacturing (AM) technique that incorporates intelligent materials into the primary framework of materials used for creating 3D printed structures or components [70]. 3D printing refers to the process of premodeled designs along with the creation of a completed product. On the other hand, the concept of 4D printing is centered around the integration of the product's design into a flexible, smart material. This is done utilizing 3D printing technology [20]. The characteristics of these objects, including color, size, and form, are subject to alteration due to changing environmental factors and triggers. These changing environmental may include PH levels, water, and temperature fluctuations [66]. 4D bioprinting provides significant benefits, chief among which is the ability of the created bio-structures to modify their functions [20,71].

According to Javaid M, Haleem A's article [72] some of the advantages of 4D printer compared to 3D printer are: smart product printing, innovate, self-assembly and the shape of the product is changed if needed.

To be successful, you need to precisely build a framework that replicates natural bone tissue. This is crucial because the framework has a direct impact on the spatial organization of cultured cells and interactions among multiple cells within the structure, causing various cellular responses to be modulated [73]. To successfully stimulate bone regeneration, it's necessary to create a functional scaffold. This involves the combination of both organic and inorganic components, such as collagen and HA, as well as the integration of microscale capillary tubes [74]. Additionally, the successful growth of denser bone tissue (400 lm) necessitates a structure with adequate blood supply, because of oxygen and nutrient diffusion constraints. Therefore, for effective reconstruction of bone tissue, it's imperative that the scaffolds possess a structural design capable of stimulating efficient blood vessel formation [75].

Utilizing a 4D printing methodology, Hwangbo, H. and colleagues introduced a novel design blueprint for a bone tissue-specific structure. The mechanism involved the construction of microscale struts embedded with tens-of-micrometers-sized channels. The goal was to replicate the hierarchically porous structure of bones which enhances osteogenic and angiogenic activities. To materialize this aim, Type I collagen was employed; this is due to the fact that the extracellular matrix (ECM) hydrogel containing it is completely biocompatible with the regrowth of bone tissue [76]. The micro channeled collagen (MC) scaffold that was created underwent further handling with simulated body fluid (SBF). This led to the creation of a scaffold surface that incorporates Hydroxyapatite (HA), and this enhancement improves its osteogenic functions. Our assessment of this complex bone-mimicking scaffold was done with the goal of achieving a functional scaffold. This would efficiently stimulate osteogenesis through human adipose stem cells (hASCs) grown under in vitro conditions [75].

A porous bone scaffold composed of polylactide (PLA)/15 wt% hydroxyapatite (HA) was also created using a direct heating 4D printer that enabled shape memory [77]. The resulting structure was highly porous, with all pore spaces being open and interconnected. Notably, complete shape recovery was exhibited in all the samples, making them ideal for use as self-fitting tissue engineering scaffolds.

**Fig. 2:**
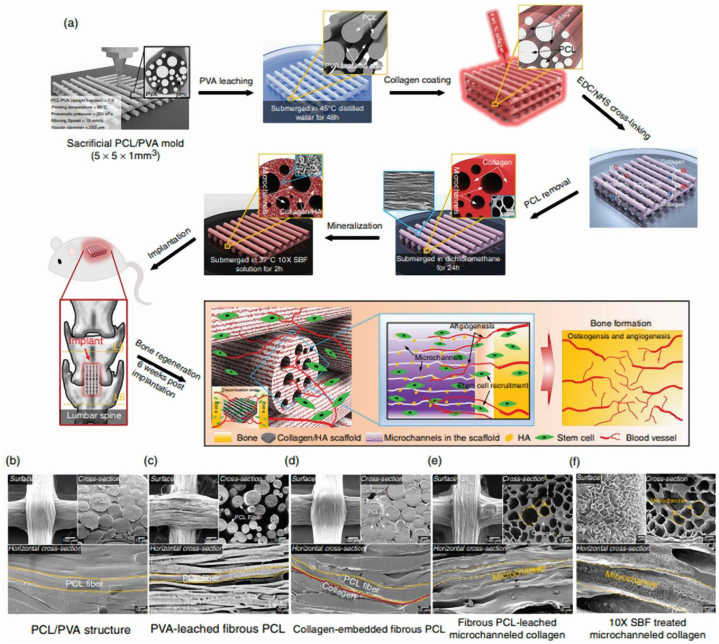
a) An image of the general process of studying the construction of hydroxyapatite scaffolds for bone repair and increasing the speed of angiogenesis and ossification. b) PCL/PVL structure. c) Leaching PVA with fibrous PCL. d) Embedded collagen with fibrous PCL. e) Collagen microchannels after leaching fibrous PCL. f) SBF treated micro channeled collagen [75].

In study by Hwangbo et al. [75] spinal fusion surgery is performed to join two or more vertebrae to stop movement that may cause pain. An alternative is tissue engineering constructs that mimic the structure and function of bone. The authors developed a layered scaffold that combines type I collagen for flexibility and hydroxyapatite for bone stiffness and conductivity. The use of 4D printing technology allows the scaffold to be printed in a flat shape and, after hydration, become curved to match the curvature of the spine. This will better integrate the implant into the spine. The scaffold supported the growth of human bone marrow stem cells and the formation of a new bone matrix in vitro. When implanted in a mouse model, new bone tissue formed throughout the scaffold in 8 weeks, demonstrating its potential for spinal fusion. Fig. 2a shows a picture of the general process of studying the construction of hydroxyapatite scaffolds for bone repair, and the effect of this scaffold on the speed of angiogenesis and ossification. Fig. 2b shows the PCL/PVL structure. Fig. 2c shows the step of washing PVA with fibrous PCL. Fig. 2d shows collagen embedded with fibrous PCL. Fig. 2e shows collagen microchannel after washing the fibrous PCL. Fig. 2f shows the treated microchannel collagens with SBF.

### 4.1 Methods and stimuli related to make scaffolds with 4D printer

**Fig. 3:**
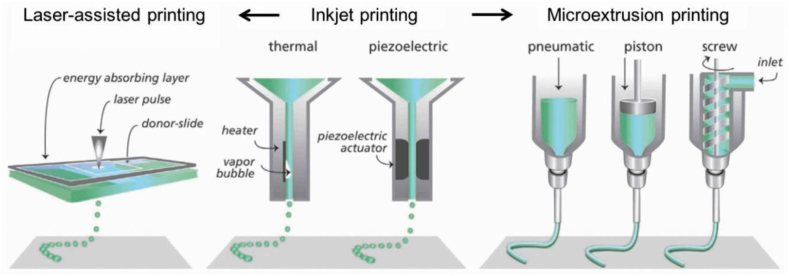
Some methods of printing additive materials with 4D printers [82].

In general, the method and methods used for printing with a 4D printer are similar to 3D printers. For utilized 4D printing there are various categories of AM technologies (Fig. 3) [[78], [79], [80], [81]]. They are distinguished based on how they deposit ink or materials (Table 1).

**Table 1:** 4D printer methods for making scaffolds.

Extrusion-based	Ref
Fusion deposition modeling (FDM)	[78]
Direct ink writing (DIW)	[81]
Inkjet	[80]
Vat Photopolymerization	
Stereolithography (SLA)	[79]
Digital light processing (DLP)	[79]

Fabricated structures' form and purpose can be altered based on one or several stimuli [[83], [84], [85]]. Stimuli can be classified into two groups: external and internal (Table 2).

**Table 2:** Some stimuli to stimulate smart add-ons in 4D printers.

External stimuli	Ref
Water	[85]
Temperature	[85]
Electric field	[84]
Magnetic field	[84]
Light	[83]
Internal stimuli	
Cell traction	[85]

## 5 Drug delivery with hydroxyapatite based composites

Hydroxyapatite (HA), as a drug transporter, exhibits promising traits such as simple modification, biological compatibility, appropriate dimension, and interactive surface. The evolution of 3D printing technology has brought to light the unique drug delivery system (DDS) ability of 3D printed porous HA structures to control the release of various bioactive substances for bone restoration. It is crucial to note that several influences, including microstructure, specific surface area, and coatings, may impact the effectiveness of drug load on HA frameworks [42,[86], [87], [88]].

Generally, it has been observed that drug release from hydroxyapatite (HA)-based drug delivery systems exhibits a significant initial burst during the first 24 h, sometimes releasing the entire quantity of the drug molecules. Uskokovi'c et al. sought to minimize this swift liberation of the clindamycin antibiotic from HA. They adopted a strategy of coating the apatite surface with a chitosan polymer. The findings indicated that the inclusion of the polymer curbed the accelerated release of the antibiotic over the initial 24-h period [17].

Deng et al. [89] report these nanoparticles exhibited a hexagonal shape, with an average diameter of roughly 110 nm. Hydroxyapatite nanoclusters were found to be successful vehicles for loading doxorubicin with high efficiency. The antitumor effects of the doxorubicin-loaded hydroxyapatite nanoclusters (DOX-HAP) were assessed in a colorectal cancer organoid model. DOX-HAP nanoclusters displayed favorable absorption properties in vitro studies of HCT116 colorectal cancer cells. The drug release kinetic studies revealed a continuous release of doxorubicin from these nanoparticles over a span of 96 h. Further examination via confocal imaging showed considerable penetration and buildup of DOX-HAP within multiple layers of cancerous organoids. However, this phenomenon was not replicated in normal colon organoids. The nanoclusters demonstrated a significant potency in instigating apoptosis and putting a stop to the growth of cancer organoids. Furthermore, these nanoclusters exhibited diminished toxicity to normal colon organoids compared to cancerous ones.

### 5.1 4D printing drug delivery

In general, the materials used in 4D printing include hydrogels and polymers with shape memory properties, which differ in their ability to change after printing. The degree of swelling of hydrogels depends on internal properties such as cross-linking density, micro-structural anisotropy and hydrophilicity. Specifically, hydrogel printability significantly influences both the manufacturing process selection and end-product [90].

The primary benefit of utilizing hydrogels lies in their biocompatibility and straightforward printing process using direct ink. This can be exemplified when utilizing printing methods such as DIW or FDW, wherein they necessitate not just shear stress but also a particular yield strength. Hydrogel, a simple-to-synthesize compound boasting high biocompatibility, provides a slew of other advantages. Some of these include its ability to be fine-tuned, its high performance, and its cost-effectiveness. Owing to these beneficial features, hydrogel stands as an encouraging interface material applicable in several areas of biomedical technology. These include use in implants, systems for drug delivery, as well as non-invasive diagnostics [91].

Shape memory polymers, which are highly sensitive and responsive to numerous factors leading to a change in form, have been instrumental in the advent of new technologies across several domains, notably in healthcare. The rise of intelligent materials capable of responding to biological markers and disease-related irregularities in the body, has indeed made 4D bioprinting for drug delivery a tangible reality. In the realm of biomedical applications, hydrogels are extensively used due to their strong biocompatibility and flexibility [92].

### 5.2 Responsive drug delivery systems

Intelligent drug delivery systems can be sensitive to various factors such as temperature, pH, light, electric and magnetic waves, etc. And under these factors, they can change the shape in a reversible or irreversible way and cause drug release. But in general, in molecular biology it has been adopted to produce changeable materials based on polypeptides responsive to pH and temperature (natural body temperature).

The team of researchers extensively utilized temperature-responsive PNIPAm-based polymers. They employed these polymers for the purpose of facilitating the attachment of biopolymers and cells to various surfaces [93,94].

Dai et al. [95] introduced an innovative technique that employs a heat-sensitive hydrogel (pluronic F127 diacrylic macromolecule) as a shape memory hydrogel, triggered by near-infrared light. This composite material's photosensitivity is enhanced by the inclusion of graphene oxide. A mere 240 s of exposure to near-infrared light is sufficient to return the deformed hydrogel to its initial form. The varying surface area due to the shape alterations of the structure is the key factor influencing the drug's release speed. Consequently, when the provisional form is distorted, the surface area reduces, leading to a slower rate of drug release.

#### 5.2.1 PH-responsive drug delivery systems

pH-responsive smart hydrogels are an innovational and promising form of drug delivery mechanisms. They are engineered to react to alterations in bodily pH levels, enabling a regulated release of medication on demand. The unique design allows these hydrogels to swell or contract based on changes in pH, which in turn controls the drug discharge. This aspect makes these advanced systems potentially viable for direct therapeutic applications aimed at conditions such as cancer, inflammation, and infection - which display diverse pH levels compared to healthy tissues. A different study introduced a drug delivery method employing DLP technology. In this system, the drug release was influenced by pH levels and shape-induced swelling. It was shown within this research that the regulation of drug release could be manipulated by controlling pH levels and surface area. This effectively showcased the capacity of 3D printing technology to intensify the efficacy of traditional solid dosage [96].

In pH-responsive hydrogels, the principal factor governing volume alteration is the internal hydrogen ion concentration in correlation with pH fluctuations. The pH spectrum in the human body is extensive, ranging from the high acidity found in the stomach, passing through the near neutral pH in the blood and colon, to the slightly acidic environment in the vagina. As such, pH-responsive hydrogels are extensively employed in the domain of biomedicine [97,98].

Hu and colleagues [99] studies on acrylic acid (AAc)-based hydrogels placed in alkaline and acidic environments show their swelling behavior in both conditions. At pH above 9, the carboxyl groups of AAc release protons, increasing the internal electrostatic repulsion and thus increasing the volume of the hydrogel. On the contrary, at relatively low pH, its volume shrinks. When immersed in an alkaline solution (pH > 9), the cage-like hydrogel structure swells. At the same time, the particles flow into the cage along with the liquid. In contrast, when placed in an acidic solution at reduced pH (pH < 9), the cage-like structure contracts, trapping the particles inside. By continuously adjusting the polymer system, it can eventually adapt to human physiological pH, offering prospects for potential biomedical engineering applications.

Anirudhan et al. [100] reports the development of intelligent pH-sensitive hydrogels for controlled antibiotic release. The researchers synthesized hydrogels using gelatin methacrylate and methacrylic acid. These hydrogels were loaded with two model antibiotics - doxycycline and metronidazole. The ionization of methacrylic acid at higher pH caused the hydrogels to swell, enabling PH-triggered release of the antibiotics. At gastric pH, minimal antibiotic release was observed from the hydrogels. However, at intestinal pH, there was sustained release of the antibiotics over 24–48 h. The system demonstrates promise for targeted antibiotic delivery in the treatment of bacterial infections in the intestine. Overall, the pH-sensitive hydrogels allow controlled antibiotic release in response to pathological pH.

Veselov et al. [101] report about the nanoparticles were prepared from the polymer poly(l-histidine)-poly(ethylene glycol)-biotin using an ionic gelation method. The imidazole groups of poly(l-histidine) endow the nanoparticles with pH-sensitivity, causing them to swell and enhance drug release under acidic conditions found in the tumor microenvironment. The anticancer drug doxorubicin was effectively loaded into the nanoparticles with high encapsulation efficiency. In vitro studies showed negligible drug release from the nanoparticles at physiological pH of 7.4 with sustained release of 90% of the payload occurring at tumor acidic pH of 6.8 within 60 h. Cellular uptake studies in MCF-7 breast cancer cells demonstrated higher internalization and cytotoxicity of the doxorubicin-loaded pH-sensitive nanoparticles compared to free drug. Overall, the results indicate these tumor acidity-targeting nanoparticles show promise as intelligent delivery systems for targeted chemotherapy.

In other study The micelles were prepared from the block copolymer poly(ethylene glycol)-b-poly(2-vinylpyridine) (PEG-*b*-P2VP). At physiological pH, the P2VP block is hydrophobic, allowing formation of micelles with PEG corona and P2VP core that can encapsulate drugs. However, at acidic tumor pH, the P2VP block becomes protonated and hydrophilic, leading to swelling and disassembly of the micelles. The pH-triggered release of doxorubicin from these micelles was demonstrated in vitro. The critical pH leading to micelle dissociation could be tuned by adjusting the PEG/P2VP ratio. In vivo experiments in tumor-bearing mice showed the pH-responsive micelles increased doxorubicin accumulation in the tumor compared to free drug. This enhanced the cytotoxicity and led to improved antitumor efficacy. Overall, the PEG-b-P2VP micelles represent a promising platform for pH-controlled delivery and release of anticancer drugs specifically within the tumor acidic microenvironment [102].

## 6 Materials used for 3D and 4D printing of HA-based scaffolds

### 6.1 HA-based composite/polymers

HA is a ceramic material that exhibits bioactivity, making it a promising candidate for regenerating the skeletal system. However, the utilization of the material is constrained due to its mechanical properties, predominantly its brittleness. Consequently, in an effort to enhance its stress-transmitting capacity, the incorporation of a polymer phase may be pursued, thereby augmenting strength whilst concurrently preserving the critical attribute of bioactivity [103]. HA is a commonly utilized substance with noteworthy bioactive characteristics and a substantial chemical and crystallographic likeness to the inorganic component of osseous tissue. This material finds application as an orthopedic biomaterial and in the field of dentistry for the purpose of hard tissue replacement. The characteristics of notable interest include the biocompatibility, innate ability to adhere to biological bone, porous architecture, and facilitation of nearby tissue growth [104,105]. HA possesses a unique characteristic that sets it apart from other materials commonly employed in the realm of implantology-osteoinduction. This particular phenomenon involves the stimulation of osteogenesis, thereby facilitating the formation of nascent bone tissue [106,107]. Polymers have been specifically formulated for utilization in 3D printing, and they have been deemed suitable for employment in tissue engineering applications due to a multitude of advantageous characteristics, such as their ability to enhance the tensile strength of HA-based scaffolding material.

Hydrogels represent one of the types of polymer scaffolds that show significant promise in the context of bone regeneration as a result of their numerous potential advantages [108]. The aforementioned constitute polymer chains possessing hydrophilic properties, which are arranged within a three-dimensional (3D) spatial context. The hydrophilic character of the scaffolds enables them to furnish a nourishing milieu that is conducive to the proliferation of indigenous cells. By virtue of their strong resemblance to the native extracellular matrix (ECM), these materials exhibit significant potential as vehicles for the encapsulation of drugs, cells or bioactive particles. Therefore, they act as conveyors of bioactive compounds that may enable regulated localized discharge [[109], [110], [111]].

### 6.2 HA-based composite/natural polymers

**Fig. 4:**
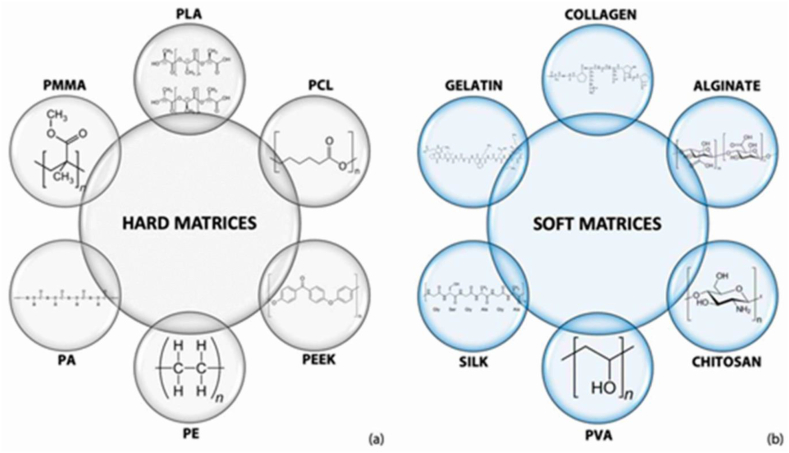
Polymeric materials find extensive application in the field of tissue engineering, particularly in combination with HA for the creation of both (a) hard/rigid and (b) soft/pliable matrices [112].

Natural polymers, namely collagen (Col), chitosan (CS), alginate (Alg), and hyaluronic acid (HyA), have been employed in bio-regenerative research, particularly in the realm of bone regeneration. Table 3 provides additional reviews of natural and synthesized polymers in composites with Hydroxyapatite. Also a multitude of synthetic and natural polymers can be effectively utilized in the reinforcement of a HA scaffold through the application of 3D printing technology for the purposes of tissue engineering, as illustrated in Fig. 4a shows some materials for making hard and rigid matrices and Fig. 4b shows some materials for creating soft and flexible matrices [112].

**Table 3:** Comprehensive account of some synthetic and natural polymers that have been recently utilized in the fabrication of biocompatible HA composite scaffolds.

Polymer and Additives	Crosslinker	Fabrication Method	In Vitro Study	In Vivo Study	Refs
Col, Zinc, Silicate	Genipin 1 wt%	3D Printing	BMSCs	Rat (critical size calvarial defect)	[113]
Alg, PVA	*cacl*2 100 mM solution	3D Printing	MC 3T3	–	[114]
PCL	–	3D Printing	Osteoblast Cell	Rat (calvarial defect)	[115]
PCL, MgO	–	3D Printing	MC 3T3-E1	–	[116]
PLA, Alg	–	3D Printing	–	–	[117]
PLA, Silk	–	3D Printing	–	–	[118]
PLA	–	3D Printing	BMSCs	–	[119]
PLA	–	3D Printing	BMSCs	White rabbits (Tibial periosteum defect)	[120]

#### 6.2.1 Collagen

**Fig. 5:**
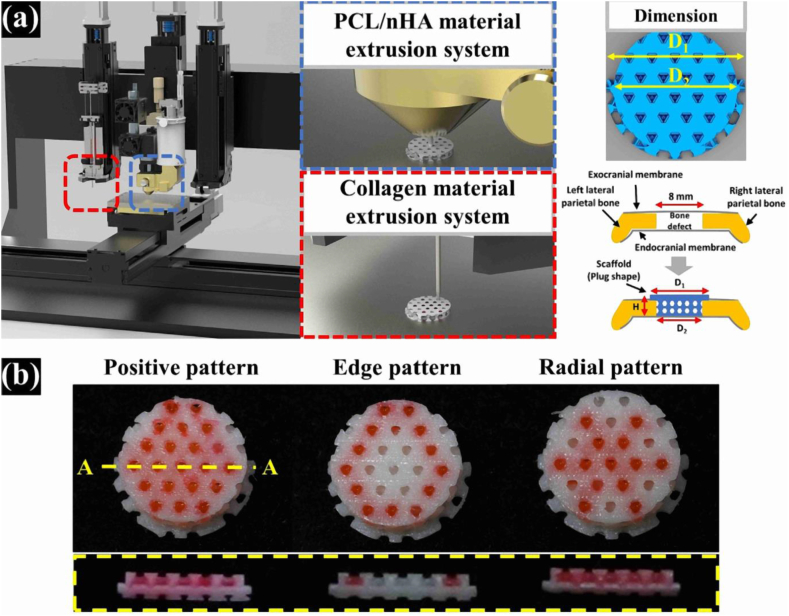
PCL/nHA/collagen scaffold. a) Extruding PCL/nHA and collagen materials by 3D printing system; b) PCL/nHA/collagen scaffolds fabricated with different collagen patterns in top and cross-sectional views. (Scale bar: 5 mm) [130].

The application of collagen sourced from different animal tissues has been utilized in various fields. The considerable biocompatibility and favorable degradability of this material have rendered it useful for numerous biomedical applications [121]. The polypeptide chain of this molecule has an abundance of glycine and proline amino acids and is organized into a secondary structure of α-helices. The helices undergo an arrangement process within tropocollagen units, comprising a triple right-handed helix, which is reinforced by both covalent and non-covalent interactions. These units, in turn, serve as the fundamental building blocks of self-assembled fibrils in collagen [122]. The primary constituents of bone tissue include type I collagen, which serves as a biopolymeric component, and HA, an inorganic component. When coalesced, a synergistic effect may occur between Col and HA, leading to an augmentation in the differentiation of osteoblasts [123,124]. Scholarly literature suggests that Col exhibits exceptional traits with regard to biocompatibility, degradation, and interaction with cellular and biomolecular constituents present in the human body [124,125]. The mechanical properties of porous HA scaffolds were enhanced through the incorporation of Col, resulting in a reduction in the overall porosity of the material [124,126]. The enhancement in the mechanical characteristics has been ascribed to the creation of intermolecular hydrogen bonds between collagen (Col) and HA, which results in an increase in the energy required to break the composition. Additionally, the intrinsic bioactivity of HA has been observed to promote osteogenic differentiation. In vitro investigations have illustrated that Col-HA biocomposites exhibit superior cytocompatibility in comparison to pure Col scaffolds. Various cell lines, including osteosarcoma [127], osteoblast [113], and fibroblast [128] cells, have exhibited enhanced attachment and proliferation when exposed to diverse concentrations of HA present in the scaffolds. An enhancement in the torsional strength of tibial defects in rabbit specimens was found to be present in those implanted with Col-HA biocomposites when compared to β-TCP controls. This observation suggests a beneficial impact of the Col-HA biocomposites on the mechanical properties of the bone [129]. The study aimed to ascertain the efficacy of a collagen I and MgHA scaffold in the treatment of osteochondral lesions through a longitudinal investigation of ten patients. The follow-up period encompassed a duration of 1–2.5 years. In contrast to prior findings from in vivo experiments conducted on animal models and clinical cohort studies, the scaffolds exhibited limited effectiveness in promoting osteochondral regeneration [122]. However, the clinical outcomes of Col-HA applications appear to be a topic of debate. In recent study of Yong et al. they created the PCL/nHA/collagen scaffold. Fig. 5 shows PCL/nHA/collagen scaffold. Fig. 5a shows that the 3D printing machine extrudes PCL/nHA/collagen material and the shape of the scaffold is plug-shaped. Fig. 5b shows PCL/nHA/collagen scaffolds fabricated with different collagen patterns in top and cross-sectional views in three patterns: positive pattern, edge pattern and radial pattern [130].

#### 6.2.2 Chitosan

Chitosan, a naturally occurring polymer, is obtained through the process of partial deacetylation of chitin under alkaline conditions. The substance in question is a copolymer composed of glucosamine and N-acetyl glucosamine, linked together to form a linear chain via β-1→4 bonds. Chitin, a polysaccharide, is notably abundant within the firm exoskeletons of arthropods, including crustaceans and insects [131]. Due to its favorable biocompatibility, biodegradability, and intrinsic antibacterial properties, chitin and its derivatives have extensive applications. Chitin-based polymers possess the capability of facilely undergoing processing procedures that enable their conversion into hydrogels or porous scaffolds. These polymers can be utilized in either their pristine state or through their chelation with several metal ions, thereby reinforcing their antimicrobial properties [132].

Li and colleagues conducted a study in which they integrated CS and HA within a scaffold possessing a hierarchical pore architecture. The scaffolds exhibited a significant increase in cell viability - 277.6% compared to the pure CS scaffold - as a result of the synergistic effect of hyaluronic acid and chondroitin sulfate (CS) [133]. The utilization of CS-HA composites in conjunction with additional biopolymeric materials has been frequently observed. A scholarly investigation conducted by Shi et al. A gradient scaffold was developed incorporating dopamine-modified Alginate (Alg), Hyaluronic Acid, and Chitosan (CS), as reported by a previous study [128]. The in vitro investigations evinced insubstantial cytotoxicity along with exceptional osteogenic activity, thus holding the potential to efficaciously stimulate bone regeneration and expedite repair of bone defects in vivo.

**Fig. 6:**
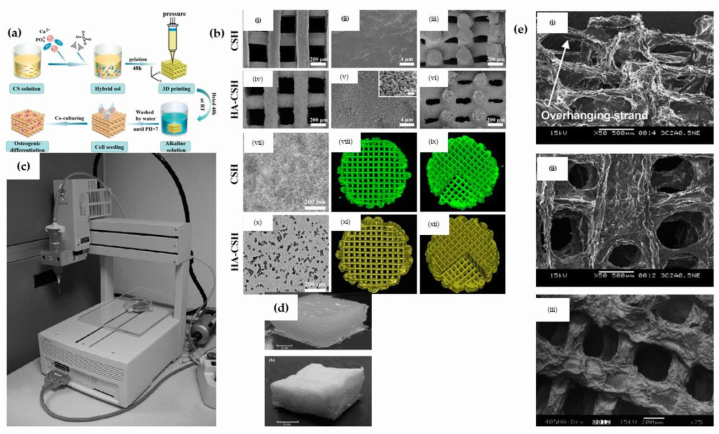
(a) Elucidating a schematic representation of the fabrication process of a hybrid scaffold comprising HA/chitosan for the purpose of bone tissue engineering (b) Examining the porosity size, morphology, and interconnectivity of chitosan and HA/chitosan scaffolds fabricated through printing techniques, with a focus on utilizing an academic style of writing (c) A rapid prototyping robotic dispensing system has been developed for the purpose of printing scaffolds comprising of HA/chitosan (d) The HA/chitosan scaffold in its printed state prior to and subsequent to the process of freeze-drying (e) SEM images of HA/chitosan scaffold [135,136].

Through the process of freeze-drying, the aforementioned researchers, Hu et al. accomplished their experimental aim. The present study has successfully devised a biomimetic hybrid scaffold, comprising hyaluronic acid, chondroitin sulfate (CS), and nanoHA (nHA). The findings indicate that the nanohybrids possess micro/nanostructures with a hierarchical design, which results in enhanced osteoblast proliferation and differentiation [134]. Ang et al. Details the development of a swift prototyping robotic dispensation mechanism for the production of composites consisting of HA/chitosan. In the course of fabrication, the bio-ink comprised of HA/chitosan was skillfully extruded through a small-caliber Teflon-coated nozzle, with an internal diameter measuring 150 μm. Fig. 6a is a description of a schematic representation of the fabrication process of the HA/chitosan hybrid scaffold for bone tissue engineering. Fig. 6b shows the examination of porosity size and morphology, and interconnection of chitosan and HA/chitosan scaffolds made through printing techniques. Fig. 6c demonstrates a rapid prototyping robotic dispensing system to print scaffolds composed of HA/chitosan by a freeze-drying process. Fig. 6d and e shows the SEM images of HA/chitosan scaffold [135].

#### 6.2.3 Alginate

Alginic acid is a biopolymer sourced from the cellular walls found in brown algae. This biopolymer comprises copolymerized glucuronic acid and mannuronic acid that are interconnected through α-1,4-glycosidic bonds [137]. Alginate substances are recognized as agents for thickening and stabilization, as they have the ability to form hydrogels that are only partially soluble in water. Biodegradable and biocompatible materials are utilized for the purposes of bone regeneration, wound healing, and the enhancement of mechanical properties [138,139].

The properties of the Alg-HA scaffold display variation depending on the mode of preparation and the proportions of alginate and HA employed. An increase in alginate concentration results in an augmented density of the scaffold accompanied by reduced porosity due to increased viscosity. This phenomenon restricts the diffusion of Alg into the pores, as established by prior research [140]. The distribution of Alg within the porous HA matrix is a result of the interaction between the Ca2+ ions present in the inorganic matrix and the COO- groups of the biopolymer. The resultant reticulation leads to a notable enhancement in the scaffold's mechanical properties, as documented in literature [139]. Alginates (Alg) coatings have been widely reported to exhibit hydrophilic properties, which consequently leads to an augmented degree of swelling and water absorption in scaffolds. However, it has been observed that when Alg is crosslinked with Ca2+ ions, the coating's hydrophilicity is mitigated, and the associated swelling is reduced. According to research conducted by Mahmoud et al. The employment of Alg-HA scaffolds was demonstrated to induce localized bone regeneration devoid of any adverse impact on the hepatic or renal functions [140]. The determination of Alg gelation and crosslinking degrees serves as essential factors in regulating the rheological attributes, particularly in terms of the printability of scaffolds through the 3D printing technique [114]. Ocando and colleagues utilized “click” chemistry to fabricate alginate and Mg-doped HA scaffolds with porous structures exhibiting dimensional hierarchy reminiscent of bone tissue. The homogeneous distribution of magnesium-substituted HA (MgHA) particles along the inner linings of the pore structures facilitates favorable adherence and growth of preosteoblastic cell populations [138].

The authors Patil et al. The current study details the production of three-dimensional porous scaffolds composed of HA coated with alginate-chitosan (Alg-CS) via wet chemical precipitation and freeze-drying techniques. The present study investigates the impact of HA content on the porosity and mechanical strength of scaffolds within a pore size range of 30–280 μm. Results demonstrate a negative correlation between HA content and porosity, concomitant with an increase in mechanical strength. The scaffolds exhibited favorable swelling behavior and biodegradability. The involvement of HA in modifying surface roughness and microtopography of the scaffold enhanced its biocompatibility, which facilitated attachment and proliferation of MG63 osteosarcoma cells in vitro. Additionally, the increased osteoblast adhesion and migration could be attributed to the favorable changes brought about by the HA coating [141].

Kohli and colleagues. The present study has amalgamated the utilization of Alg and fibrin to fabricate scaffolds that are characterized by porosity, crosslinking, and slow biodegradability while incorporating calcium phosphate. During the culture period, the MC3T3-E1 cells adhered to the scaffolds and exhibited proliferation, migration, and differentiation along the osteogenic pathway [142].

Alginate-HA (Alg-HA) scaffolds have demonstrated sound physicochemical and rheological characteristics, in addition to exceptional biocompatibility, evincing suitable cell growth and proliferation durations that render the scaffold apropos for clinical implementation. Additional research is required to further enhance the physico-chemical characteristics of the materials in question, in order to effectively position them as promising contenders in the domain of tissue engineering for bone augmentation [143].

**Fig. 7:**
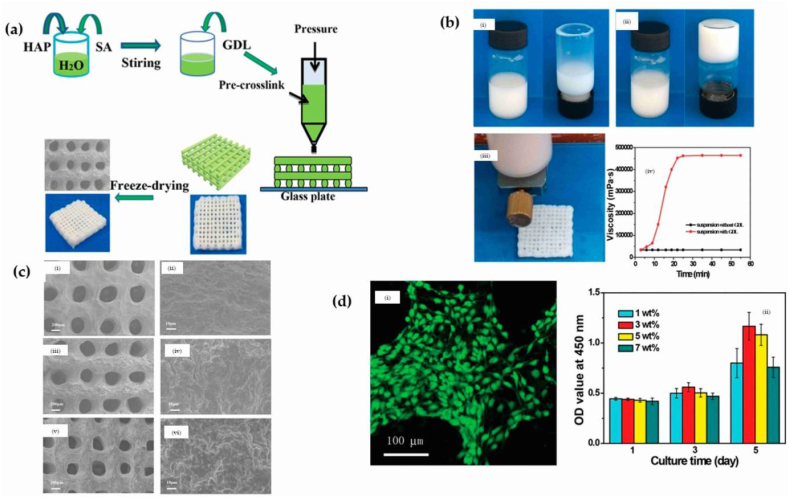
(a) Providing a schematic representation of the porous scaffolds made from a combination of HA and alginate via the extrusion-based 3D printing technique. The pre-crosslinking agent utilized in the process was d-Gluconic acid δ-lactone (GDL); (b) Utilizing photographic documentation to capture the HA/alginate suspension and resultant hydrogels formed through the utilization of GDL. The viscosity of suspensions containing HA/alginate was analyzed through the investigation of its behavior over time, while subjected to a predetermined shear rate (c) SEM images of printed porous scaffold after soaking in calcium chloride solution for different times (0, 5, and 10 h). (d) The morphology and cell proliferation of BMSCs on printed scaffolds [144].

In the research of liu et al., the pre-crosslinking of the HA/alginate nanocomposite via utilization of DGluconic acid δ-lactone (GDL) resulted in an enhanced mechanical performance of the printed HA/alginate scaffold. The adjustment of printing conditions provides a feasible means for controlling the porosity and pore configurations of the HA/alginate scaffold that has undergone printing. Throughout the print manufacturing procedure, the incorporation of curcumin, a potent anti-inflammatory agent, onto the fabricated scaffold is a feasible approach towards achieving controlled and sustained drug delivery. Furthermore, the outcomes of in vitro experimentation indicate that mouse bone mesenchymal stem cells (mBMSCs) exhibited a propensity to attach onto the permeable HA/alginate scaffolds, as illustrated in Fig. 7 [144]. In Fig. 7a, porous scaffolds made of a combination of HA and alginate are shown through the 3D printing method based on extrusion. The pre-crosslinking agent used in this process was d-Gluconic acid δ-lactone (GDL). Fig. 7b demonstrates using photographic documentation to capture HA/alginate suspensions and resulting hydrogels through the use of GDL. The viscosity of suspensions containing HA/alginate was analyzed by examining its behavior over time, while exposed to a predetermined shear rate. Fig. 7c shows the SEM images of the printed porous scaffold after soaking in calcium chloride solution. Fig. 7d shows the morphology and cell proliferation of BMSCs on the printed scaffolds, which indicates the success of this scaffold in the treatment process.

#### 6.2.4 Hyaluronic acid

Hyaluronic acid plays a pivotal role as a constituent of the extracellular matrix within the human body. Over the past few decades, bone regeneration has gained considerable popularity, particularly in the realms of craniofacial and dental medicine. Composite scaffolds that have been soaked in Hyaluronic Acid (HyA) have demonstrated impressive potential in enhancing the processes of osteogenesis and mineralization. The HyA derivatives were utilized as localized release vectors, rather than as scaffold entities, by effectively loading a variety of osteoinductive or osteogenic agents, thus achieving a controlled release. According to Kaczmarek et al. the utilization of loaded vectors that are immobilized on implant surfaces can significantly enhance osteointegration. Scaffold constructs were generated using Hyaluronic acid (HyA), Chitosan (CS), and Collagen (Col) with the addition of nano-hydroxyapatite (nHA) through the process of lyophilization. Subsequently, the biocompatibility of these constructs was thoroughly examined [145]. The findings from cell culture studies demonstrated that the incorporation of nHA into scaffolds facilitated enhanced cellular adhesion and proliferation. Conversely, in vivo examinations performed six months post-implantation on the adjacent tissue surrounding the scaffolds revealed positive outcomes in terms of wound healing and absence of any inflammatory response to the implants. The incorporation of nHA into the Hyaluronic Acid/Chondroitin Sulfate/Collagen (HyA/CS/Col) scaffolds resulted in the retardation of the implant biodegradation process, thereby conferring enhanced stability to the scaffold when exposed to adjoining tissues [146]. Yang and colleagues. In this study, an injectable HyA-Alg hydrogel system was devised and incorporated with exosomes, which are nanovesicles that are endogenously produced by cells, to address bone defects in rats in vivo. The results obtained highlighted the promising capacity of this approach to facilitate bone defect regeneration [147].

The investigation carried out by Sujana et al. in their research is an instance of suitable academic writing. The present study involved the development of biocompatible nanofibers composed of Hyaluronic Acid (HyA), Poly (l-lactic acid)-*co*-poly(ε-caprolactone) (PLCL), Fibroin, and Hyaluronan by means of the electrospinning technique [148]. The objective was to mimic the structure of the native Extracellular Matrix (ECM) for potential biomedical applications. The nanofibrous scaffolds exhibit increased porosity when compared to their micro-sized fiber counterparts, thereby enabling efficient exchange of vital nutrients and metabolic byproducts. The proliferation level of osteoblasts cultivated on the aforementioned scaffolds exhibited a 53% increase in comparison to their microfibrous counterparts. Moreover, the inclusion of bioactive molecules within the scaffolds led to a 63% higher degree of osteogenic differentiation and mineralization, thereby demonstrating their exceptional suitability as biocomposites for the purpose of bone tissue engineering [148].

#### 6.2.5 HA-based composite/ceramics

Ceramic materials possess a composite structure that comprises of metallic and nonmetallic components, rendering them an ideal option for 3D printed scaffold fabrication, which necessitates robust mechanical properties and biocompatibility [149]. Ceramic materials possess the potential for scaffold fabrication to facilitate bone regeneration as a result of their exceptional apatite-mineralization properties [150]. HA, a well-known ceramic material, is a prevalent constituent of the human dentition and skeletal system [151]. Consequently, the utilization of HA, or analogous ceramics, has become an appealing option for fabricating scaffolds possessing robust mechanical characteristics akin to those of authentic bone. HA has generated considerable interest within the realm of regenerative medicine and has, therefore, been widely utilized as a fundamental material for the fabrication of 3D printed scaffolds [152]. The interconnected channels with a porosity of 500 μm within the synthesized scaffolds demonstrated the capacity to stimulate cellular proliferation of mouse MC3T3-E1, highlighting the potential of HA scaffolds for bone regeneration [36]. A recent investigation employed a rapid prototyping approach utilizing computer-assisted 3D printing to produce scaffolds composed of HA and tricalcium phosphate (TCP). Scaffolds were fabricated through a method of layer-by-layer deposition utilizing HA and tricalcium phosphate, with a subsequent process of sintering [153]. The scaffolds were seeded with human osteoblasts that had been isolated from the cancellous bone of the human iliac crest. The resulting scaffold demonstrated a notable degree of biocompatibility, in conjunction with minimal observed cytotoxicity. The present findings substantiate the notion that HA materials exhibit biocompatibility and possess the capacity to support cell proliferation and survival [36]. The utilization of a composite of ceramic materials in the manufacturing process of 3D printed scaffolds warrants further examination as it has the potential to generate materials possessing a significantly enhanced level of precision in their design, appropriate compressive strength, and enable the promotion of cellular proliferation and differentiation. Such developments can be implemented to address the requirements of both load bearing and non-load bearing applications [36].

Indeed, the creation of an appropriate scaffold capable of forming bone, requires specific porosity and unique morphology. This aids in facilitating cell interconnection, cell adhesion, cell movement, and eventually, the formation of bone (osteogenesis) [154]. HA-polymer composite scaffolds are frequently employed in the realm of tissue engineering and regenerative medicine owing to their notable biocompatibility and mechanical characteristics. The following provides a some of them to scaffolds composed of HA-polymer composites.

#### 6.2.6 Poly (lactic-*co*-glycolic acid) (PLGA)/HA composite scaffold

PLGA is known for its biocompatibility and favorable mechanical properties [155,156]. PLGA is made of two polymers PLA and PGA. By considering and adjusting the percentage of these two polymers in PLGA, the rate of hydrolysis and decomposition can be controlled so that the more PGA in the structure, the faster the rate and process of decomposition and hydrolysis of this copolymer. The final products of hydrolysis include lactic acid and glycolic acid, which are part of the natural metabolic products of the human body and can be easily excreted through urine [157,158]. PLGA, as a biodegradable polymer, has gained significant attention in the field of tissue engineering due to its extensive utilization in various medical applications. The conjoining of HA and a material substantiates a compound scaffold which bears semblance in both structure and functionality to the innate human bone [159].

In the discussion of tissue engineering, the good and successful performance of this copolymer was investigated by Norouzi et al. in the discussion of skin tissue [160], and Lee et al. in the discussion of cartilage tissue [161]. Also, in the discussion of bone tissue engineering, Sheikh and his colleagues stated that for bone tissue engineering and repairing its defects, one should learn the bone structure in order to understand concepts such as physical strength and the formation of new tissue. In their research, they conducted an examination on the three-dimensional PLGA scaffold enhanced with hydroxyapatite nanoparticles. Their findings documented several positive results including an increase in mechanical strength, enhanced hydrophilicity, and improved osteoblast survival [162]. Lai and his team fabricated a porous scaffold composed of PLGA, TCP, and Mg. Their aim was to speed up the bone regrowth process in cases of steroid-related osteonecrosis. Their findings revealed that these specifically designed PLGA/TCP/Mg scaffolds substantially improved the formation of new bones, increased blood vessel development, and blood circulation in rabbit models suffering from steroid-induced osteonecrosis [163].

It's possible to control drug loading and release by incorporating drugs into a printable vehicle matrix. This approach was executed by Shim and colleagues who utilized a compound of PCL and PLGA for the creation of scaffolds. They introduced a substance called rhBMP-2, enclosed within a combination of collagen and gelatin hydrogels, into the PCL/PLGA scaffold using a multi-head deposition system. They noticed a controlled release of rhBMP-2 over a period of up to 28 days. This method resulted in an accelerated healing process of critical sized bone defects in rabbit models. Additionally, it was able to prevent the inflammatory responses usually associated with burst release [164].

A new version of porous HA scaffold containing PLGA microsphere accompanied with Dexamethasone to deliver inorganic calcium phosphate in order to generate bone tissue in vivo [165,166].

#### 6.2.7 Chitosan/HA (CS/HA) composite scaffold

Chitosan is a naturally occurring polymer originating from chitin, a constituent of shells of crustaceans. The amalgamation of this substance with HA results in the formation of a composite scaffold that exhibits exceptional biocompatibility and biodegradability [167]. Major substances examined for biomedical applications include Hydroxyapatite (HA) and Chitosan (CS) biopolymer. In the orthopedic domain, these constituents are instrumental, serving as replacement for bone tissues or mechanisms for drug delivery. However, when used in isolation, the durability of Hydroxyapatite is considerably delicate, and Chitosan's mechanical strength is notably feeble [168]. When integrated into the structure of various inorganic materials, this polymer can boost osteoblast cell proliferation, thus aiding in the healing of bone fractures [169].

As mentioned before, in addition to these, chitosan can also be used as a suitable polymer coating for the controlled release of drugs [17].

#### 6.2.8 Polyethylene glycol (PEG)/HA composite scaffold

Polyethylene glycol (PEG) is a hydrophilic polymer that exhibits the potential to enhance both the mechanical properties and biocompatibility of HA scaffolds. The resultant composite scaffold demonstrates potential utility in the context of bone tissue engineering endeavors [170].

#### 6.2.9 Polycaprolactone (PCL)/HA composite scaffold

**Fig. 8:**
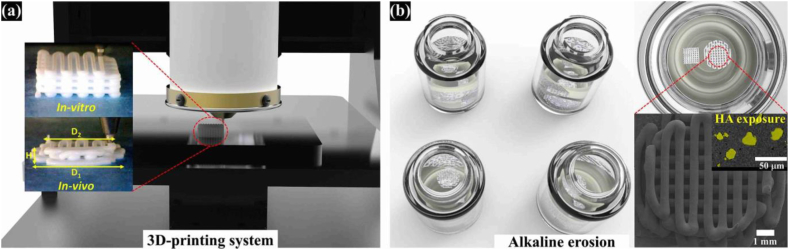
Images of 3D printed scaffolds exposed to HA a) 3D Print system b) Alkaline corrosion [115].

The biodegradable polyester, PCL, has been utilized in diverse tissue engineering contexts. When amalgamated with HA, it generates a composite structure which can be implemented for the purpose of bone regeneration [171]. In Fig. 8, Yong et al. showed that the combination of HA and polylactic acid achieved good success in terms of scaffold construction and in-vivo and in-vitro tests [115]. Fig. 8a shows the 3D printing system for scaffold construction and Fig. 8b shows the alkaline corrosion of the scaffold, which results in the biocompatibility of the scaffold for bone tissue engineering.

#### 6.2.10 Gelatin/HA composite scaffold

Gelatin is a naturally occurring polymer extracted from collagen, a primary constituent of the extracellular matrix presents in several tissues. The conflation of HA and this substance produces a composite scaffold with noteworthy potential for utilization in bone tissue engineering endeavors [172].

## 7 Conclusion and perspective

### 7.1 Conclusion

To summarize, the domain of 3D printing HA-polymer composites for bone tissue engineering demonstrates considerable potential. The literature examined underscores the potential of this technology to fabricate scaffolds possessing customized structures, mechanical properties, and bioactivity. However, there are challenges that need attention in order to achieve an optimal pore architecture, enhance cell-material interactions, and ensure long-term stability. Advancements in technology have led to the prospect of utilizing HA-polymer composites created through 3D printing in the field of bone tissue engineering. This provides the means for revolutionary potential and contributes to the advancement of efficacious regenerative therapies. Four-dimensional (4D) bioprinting heralds a pioneering epoch in tissue engineering, where dynamic structures capable of adapting and evolving through time are created. By integrating temporality as a fourth dimension, 4D bioprinting engenders scaffolds and tissues with innate shape-morphing capabilities, thus emulating the dynamic attributes of native human tissues more faithfully. The cardinal distinction of 4D bioprinting lies in the incorporation of “smart” biomaterials that can respond to physiological cues or external triggers. Shape memory polymers, hydrogels, and other novel biomaterials are engineered to transform or self-assemble on-demand, empowering printed tissues to metamorphose from one intricate architecture to another. This enables researchers to have unparalleled control over the spatiotemporal exposure of biological factors within a tissue scaffold, thereby directing cell behavior and tissue maturation. With its inherent capacity for self-evolution, 4D bioprinting constitutes an enabling platform to replicate the elaborate dynamism of the human body's indigenous tissues and organs. For example, harnessing 4D bioprinting principles to create tissues with inbuilt vasculature that can generate new blood vessel networks under hypoxic conditions, or bone tissue scaffolds that remodel and regenerate analogously to the natural skeletal system.

### 7.2 Perspective

Looking ahead, advanced 4D bioprinting techniques integrating stem cell science, developmental biology, and AI-driven tissue growth modeling hold tremendous potential for propelling next-generation tissue engineering. 4D bioprinting is bringing us closer to customizable, autonomous, and intelligent organ systems, enabling transformative approaches in regenerative medicine and in vitro disease modeling. On the other hand, most existing materials only respond to a single stimulus, which poses a limitation if the stimulus-producing equipment fails. Therefore, it is crucial to develop materials that are responsive to multiple stimuli. The combination of various types of responsive materials in the future is expected to bring outstanding breakthroughs to 4D printing. In addition, for 4D bioprinting, ideal materials require good biocompatibility and proper mechanical properties. While most biomaterials currently used exhibit good biological compatibility, their mechanical properties have not yet been thoroughly tested. Although various stimuli-responsive microstructures have been reported, the embryonic stage of 4D printing demands significant efforts, including the development and improvement of new materials and printing methods. Despite the outlined challenges, similar to other emerging technologies, 4D bioprinting is expected to have a significant impact and promising prospects for practical applications in the near future.

## Author contribution statement

All author listed have significantly contributed to the development and the writing of this article.

## Data availability statement

Data will be made available on request.

## Declaration of competing interest

The authors declare that they have no known competing financial interests or personal relationships that could have appeared to influence the work reported in this paper.
